# Galectin-2 Induces a Proinflammatory, Anti-Arteriogenic Phenotype in Monocytes and Macrophages

**DOI:** 10.1371/journal.pone.0124347

**Published:** 2015-04-17

**Authors:** Cansu Yıldırım, Daphne Y. S. Vogel, Maurits R. Hollander, Josefien M. Baggen, Ruud D. Fontijn, Sylvia Nieuwenhuis, Anouk Haverkamp, Margreet R. de Vries, Paul H. A. Quax, Juan J. Garcia-Vallejo, Anja M. van der Laan, Christine D. Dijkstra, Tineke C. T. M. van der Pouw Kraan, Niels van Royen, Anton J. G. Horrevoets

**Affiliations:** 1 Dept of Molecular Cell Biology and Immunology, VU University Medical Centre, Amsterdam, the Netherlands; 2 Dept of Cardiology, VU University Medical Centre, Amsterdam, the Netherlands; 3 Dept of Vascular Surgery, Einthoven Laboratories, Leiden University Medical Centre, Leiden, the Netherlands; 4 Dept of Cardiology, Academic Medical Centre, University of Amsterdam, Amsterdam, the Netherlands; Gustave Roussy, FRANCE

## Abstract

Galectin-2 is a monocyte-expressed carbohydrate-binding lectin, for which increased expression is genetically determined and associated with decreased collateral arteriogenesis in obstructive coronary artery disease patients. The inhibiting effect of galectin-2 on arteriogenesis was confirmed *in vivo*, but the mechanism is largely unknown. In this study we aimed to explore the effects of galectin-2 on monocyte/macrophage phenotype *in vitro* and *vivo*, and to identify the receptor by which galectin-2 exerts these effects. We now show that the binding of galectin-2 to different circulating human monocyte subsets is dependent on monocyte surface expression levels of CD14. The high affinity binding is blocked by an anti-CD14 antibody but not by carbohydrates, indicating a specific protein-protein interaction. Galectin-2 binding to human monocytes modulated their transcriptome by inducing proinflammatory cytokines and inhibiting pro-arteriogenic factors, while attenuating monocyte migration. Using specific knock-out mice, we show that galectin-2 acts through the CD14/toll-like receptor (TLR)-4 pathway. Furthermore, galectin-2 skews human macrophages to a M1-like proinflammatory phenotype, characterized by a reduced motility and expression of an anti-arteriogenic cytokine/growth factor repertoire. This is accompanied by a switch in surface protein expression to CD40-high and CD206-low (M1). In a murine model we show that galectin-2 administration, known to attenuate arteriogenesis, leads to increased numbers of CD40-positive (M1) and reduced numbers of CD206-positive (M2) macrophages surrounding actively remodeling collateral arteries. In conclusion galectin-2 is the first endogenous CD14/TLR4 ligand that induces a proinflammatory, non-arteriogenic phenotype in monocytes/macrophages. Interference with CD14-Galectin-2 interaction may provide a new intervention strategy to stimulate growth of collateral arteries in genetically compromised cardiovascular patients.

## Introduction

Galectins form a family of beta-galactoside binding proteins with a wide cell- and tissue distribution [1–5]. Members of the galectin family can bind to distinct cell surface- or extracellular matrix glycoconjugates [1,4] and thus play a role in cell activation, cytokine secretion, adhesion, migration and apoptosis [3]. Intracellular, galectins may affect signaling pathways through interaction with cytoplasmic- and nuclear proteins, in a carbohydrate-independent manner [1,5].

Galectins have important roles in immune responses, autoimmune diseases, cancer, atherosclerosis, neuronal degeneration, and wound healing [1,4,6–9].

Recently, results of our group showed involvement of Galectin-2 in arteriogenesis in patients with ischemic heart disease. During arteriogenesis, collateral vessels undergo remodeling to increase their diameter, thus compensating for upstream stenosis. A well-developed coronary collateral circulation is protective in patients with ischemic heart disease [10]. We demonstrated that galectin-2 mRNA expression is increased in both monocytes and macrophages of coronary artery disease (CAD) patients with a low arteriogenic response and that galectin-2 impairs arteriogenesis *in vivo* in a murine model [11]. Interestingly, galectin-2 treatment reduced the number of perivascular macrophages, suggesting that galectin-2 may inhibit arteriogenesis by modulating monocyte/macrophage responses [11].

The local tissue microenvironment largely governs specific macrophage responses and polarization to either M1 or M2 subtypes. Interferon-γ (IFN-γ) and tumor necrosis factor-α (TNF-α) are important inducers of classical activation to M1 macrophages. M1 macrophages produce high levels of proinflammatory cytokines, reactive oxygen and nitrogen radicals and fuel Th1 responses.IL-4 and IL-13 induce alternative activation to anti-inflammatory M2 macrophages that are involved in Th2 responses, tissue repair, remodeling, and (tumor) angiogenesis [12–16]. Accordingly, M1 and M2 macrophage subtypes express profoundly different repertoires of cytokines, chemokines and receptors [17–22]. In the present study, we have investigated the mechanism through which galectin-2 exerts its anti-arteriogenic effect on both monocytes and macrophages by examining the changes in macrophage phenotype and physiology. We show for the first time that galectin-2 polarizes monocytes and macrophages to a proinflammatory M1 state, while preventing pro-arteriogenic M2 differentiation.

## Materials and Methods

### Galectin-1 and -2 expression and purification

The open reading frames of recombinant human- and mouse galectin-2 with a 6xHis tag inserted at the N-terminal end were synthesized and optimized for E.Coli codon usage by BaseClear (Leiden, The Netherlands). For production in *Escherichia coli* BL21 (DE3), the sequences were cloned into the *N*-Gal-1 expression vector (provided by Prof. Gabriel A. Rabinovich, University of Buenos Aires, Buenos Aires, Argentina and Dr. Juan M. Ilarregui, VUmc Amsterdam). Human galectin-1, human—and mouse galectin-2 expression was induced in the cells by 1 mM isopropyl-β-D-thiogalactopyranoside (VWR International, Amsterdam, The Netherlands). Bacteria were lysed with Bugbuster protein extraction reagent (EMD Chemicals, San Diego, CA, USA) supplemented with protease inhibitor cocktail (Roche Diagnostics, Almere, The Netherlands) according to the manufacturer's instructions. To remove insoluble cell debris, the cell lysate was centrifuged using Sorvall SS34 centrifuge at 16.000 x g for 20 minutes at 4°C. Galectins were purified from the supernatant by affinity chromatography on Ni-NTA agarose beads (Qiagen, Venlo, The Netherlands). The column was then extensively washed with a buffer containing 50 mM Tris, 150 mM NaCl, 20 mM Imidazole, 10% Glycerol, and 4 mM β-mercaptoethanol (pH 8.0), and eluted with the same buffer containing higher concentration of imidazole (300 mM). Finally, the protein was desalted using PD-10 columns (GE Healthcare Life Sciences, Uppsala, Sweden) in storage buffer containing 50 mM Tris, 150 mM NaCl, 10% Glycerol, and 4 mM β-mercaptoethanol (pH 8.0). Galectin purity was analyzed by SDS-PAGE on a 15% polyacrylamide gel, and the protein was stained with Coomassie blue stain (Merck, Darmstadt, Germany). After production and purification, both human and mouse galectin-2 appeared as a single homogenous protein band with a molecular weight of 15 kDa. Human galectin-1 appeared as a dimer with a molecular weight of 28 kDa, and as a monomer of 14 kDa (S1 Fig). Galectin concentration was measured using NanoDrop ND-1000 Spectrophotometer (Thermo Scientific, Breda, The Netherlands) and stored at -80°C until use.

### Human monocyte isolation, culture and *in vitro* polarization to macrophage subtypes

Peripheral blood mononuclear cells (PBMCs) were isolated from anonymized healthy volunteer buffy coats (Sanquin, Blood bank, Amsterdam, The Netherlands), using Ficoll density gradient (Lymphoprep™, Axis-Shield, Oslo, Norway). For the isolation of monocytes we performed a second density gradient step using 150 x 10^6^ PBMCs overlayed on Percoll solution (GE Healthcare Life Sciences), and centrifuged at 400 x g for 40 min at RT. After centrifugation, monocytes were collected and washed once in phosphate buffered saline (PBS) containing 1% citrate (Sigma-Aldrich, Zwijndrecht, The Netherlands) at 400 x g for 10 minutes and twice at 277 x g for 5 minutes. Monocytes were cultured in RPMI 1640 medium (Invitrogen, Bleiswijk, The Netherlands) containing 10% heat-inactivated fetal bovine serum (FBS; Lonza, Breda, The Netherlands), 1% penicillin/streptomycin (Lonza) and 2 mM L-glutamine (Lonza), in tissue-culture plates (Greiner Bio-One, Alphen a/d Rijn, The Netherlands) at a density of 1 x 10^6^/ml. The monocytes were incubated for three hours at 37°C and 5% CO_2_ with indicated concentrations of recombinant human and mouse galectin-2 or human galectin-1, 20 mM lactose anhydrate (Sigma-Aldrich), 10 ng/ml LPS (from *E*. *coli* 0111:B4; Invivogen, Toulouse, France), 20 μg/ml polymyxin B (PMB; Sigma-Aldrich), 1 μg/ml peptidoglycan (PGN; Sigma-Aldrich) or 20 μg/ml anti-human CD14 blocking antibody (Invivogen, Toulouse, France).

For macrophage differentiation, the monocytes were seeded at a density of 1 x 10^6^/ml in DMEM complete medium (Invitrogen) supplemented with 5% heat-inactivated human AB serum (Sanquin, Blood bank), 1% penicillin/streptomycin and 2 mM L-glutamine. The macrophages were differentiated by culturing them at 37°C with 5% CO_2_ for six days in 145 mm petridishes (Greiner Bio-One).

After six days of culture, the adherent macrophages (M0) were harvested with 4 mg/ml lidocaine hydrochloride monohydrate (Sigma-Aldrich) in PBS at 37°C and 5% CO_2_ for 10 minutes.

Macrophages were seeded at a density of 1 x 10^6^/ml in 6 well tissue-culture plates (Greiner Bio-One) in DMEM complete medium and incubated with 10 ng/ml rhIL-4 (Immunotools, Friesoythe, Germany) for differentiation into alternatively activated M2 macrophages for 48 hours. To obtain classically activated M1 macrophages, cells were incubated with 100 U/ml rhIFN-γ (U-Cytech, Utrecht, The Netherlands) and after 7 days of culture, 10 ng/ml LPS (Ultra pure from *E*. *coli* 0111:B4; Invivogen) was added and incubated for another 24 hours for full differentiation into M1 macrophages. Recombinant human galectin-2 (10 μg/ml) or storage buffer (vehicle control) was also added at day 7 for the last 24 hours. Control macrophages (M0) were cultured for the same period in DMEM complete medium without additional stimuli. The different macrophage subtypes were used for further experiments as indicated. Phenotypical and functional characterization of macrophages was performed at day 8.

### Galectin binding assays

Galectin binding to human monocytes, human monocyte-derived macrophages and-dendritic cells, human T-cells, and mouse macrophage cell line RAW 264.7 was performed. To that end, galectins were labelled with biotin (Sigma-Aldrich) in *N*-Hydroxysuccinimide (NHS) solution (Sigma-Aldrich). The protein was purified using PD-10 columns (GE Healthcare Life Sciences) in PBS. The galectin concentration was measured using NanoDrop ND-1000 Spectrophotometer. Briefly, cells were suspended in 0.5% bovine serum albumin (BSA; Roche Diagnostics) in PBS containing the indicated amount of biotinylated galectin at 4°C for 30 minutes. For determination of the binding affinity of galectins to monocytes, graded concentrations of biotinylated galectins were used, as indicated. The cells were then washed and incubated with streptavidin-alexa fluor 488 (1:800; Invitrogen) at 4°C for 30 minutes. Background staining was determined by omitting the biotinylated lectin. The binding was also performed in the presence of indicated concentrations of lactose, thiodigalactoside (Carbosynth Compton, UK), PMB (Sigma-Aldrich), or 100 μg/ml anti-human CD14 blocking antibody (Invivogen). For analysis of different subsets of human monocytes, non-specific antibody binding was blocked with 10% normal mouse serum (NMS) in 0.5% BSA/PBS, while cells were labeled with mouse anti-human CD14-PE (Immunotools) and mouse anti-human CD16-APC antibodies (Immunotools). After washing, the cells were analyzed by flow cytometry using Cyan ADP High Performance Research Flow Cytometer (Beckman Coulter, Woerden, Netherlands). The data were analyzed using the Summit V4.3 program (Dako, Fort Collins, CO, USA).

### Human monocyte migration assay

Human monocyte migration was studied using 24-well Transwell inserts (6.5 mm) with polycarbonate filters of 5-μm pore size (Corning Life Sciences, Amsterdam, The Netherlands). Briefly, the filters were pre-coated with 10 μg/ml fibronectin (Sigma-Aldrich) in PBS for one hour at RT. Then, 0.5 x 10^6^ monocytes diluted in 100 μl of RPMI 1640 medium containing 0.5% BSA were added in the presence or absence of 50 μg/ml recombinant human galectin-2 to the upper chamber of the insert. The lower chamber contained 600 μl of RPMI 1640 medium containing 0.5% BSA without any chemokine. The plates were incubated at 37°C in 5% CO_2_ for 24 hours and monocytes that had migrated into the lower chamber were photographed using Leica DM IL microscope at 20 times original magnification (Leica Microsystems B.V., Rijswijk, The Netherlands), counted using flow cytometry by labeling the cells with a non-blocking CD14-PE antibody (Immunotools), and acquiring the cells at a constant acquisition rate over a fixed time-period.

### RNA isolation, cDNA synthesis, quantitative real-time PCR

Total RNA was isolated from human monocytes or macrophages using RNeasy mini kit (Qiagen) according to the manufacturer’s instructions, including a DNase I (Qiagen) digestion step to remove genomic DNA. RNA samples were concentrated by SpeedVac for 30 minutes. RNA purity and concentration was measured using NanoDrop ND-1000 Spectrophotometer. For cDNA synthesis, between 100–500 ng of total RNA per sample was reverse transcribed using RevertAid H Minus First Strand cDNA Synthesis Kit (Thermo Fisher Scientific, Waltham, MA, USA) following the manufacturer's instructions. Quantitative real-time polymerase chain reaction (PCR) was carried out in an ABI PRISM 7900HT system (Applied Biosystems, Foster City, CA, USA) with the following primers (Invitrogen) designed by Primer Express version 2.0 (Applied Biosystems):

**Table:** 

Human genes	Primer sequences (5'-3')
CD40	Forward: TCCAATGTGTCATCTGCTTTCG
	Reverse: TCAGTCTTGTTTGTGCCTGCC
TNF-α	Forward: CCAAGCCCTGGTATGAGCC
	Reverse: GCCGATTGATCTCAGCGC
IL-6	Forward: TGCAATAACCACCCCTGACC
	Reverse: TGCGCAGAATGAGATGAGTTG
IL12p40	Forward: CCAGAGCAGTGAGGTCTTAGGC
	Reverse: TGTGAAGCAGCAGGAGCG
IFN-β	Forward: ACAGACTTACAGGTTACCTCCGAAAC
	Reverse: CCAGTCCCAGAGGGCACAGGCTAGGAG
GAPDH	Forward: GCCAGCCGAGCCACATC
	Reverse: TGACCAGGCGCCCAATAC
CD206	Forward: CCTGTGCATTCCCGTTCAA
	Reverse: TCGTGCAATCTGCGTACCAC
MMP-2	Forward: CGAATCAGGCATCGAGACAG
	Reverse: AATGTCAGGAGAGGCCCCA
MMP-9	Forward: CTTTGGACACGCACGACGT
	Reverse: CCTGGTTCAACTCACTCCGG
TGF-β1	Forward: CAGCATCTGCAAAGCTCCC
	Reverse: AAGTCAATGTACAGCTGCCGC
VEGF-A	Forward: ACCTCCACCATGCCAAGTG
	Reverse: GGCAGTAGCTGCGCTGATAG
PDGF-B	Forward: GCAGCAGCTTCAGAGACCAAC
	Reverse: ATCGGCAGGAGAGTGTGTGG
PDGF-C	Forward: CCACGAGGTCCTTCAGTTGAG
	Reverse: CGTCGGTGAGTGATTTGTGC
HGF	Forward: GCAAGAAAACAATGCCTCTGG
	Reverse: GAGGTCAAATTCATGGCCAA
CCL26	Forward: ACCAGTAACAGCTGCTCCCA
	Reverse: TTGCACCCATTTTTTCCTTG
CCL18	Forward: CCAGGTGTCATCCTCCTAACCA
	Reverse: GCCCTCGCAGCTTCCA

Briefly, in a 10 μl reaction volume, 4 μl of diluted cDNA, 5 μl SYBR Green PCR Master Mix (Applied Biosystems), and 0.5 uM of each gene-specific primers were mixed. Gene expression levels were calculated using an arbitrary standard curve and normalized to the human housekeeping gene glyceraldehyde 3-phosphate dehydrogenase (GAPDH). Relative gene expression levels were expressed as a fold change relative to respective untreated samples unless otherwise indicated.

### Flow cytometry of human macrophages

The expression of CD40 and mannose receptor (CD206) was analyzed on different human macrophages subtypes by flow cytometry. Briefly, macrophages were detached with 4 mg/ml lidocaine hydrochloride monohydrate in PBS at 37°C, 5% CO_2_ for 10 minutes, washed once in PBS, and fixed in 4% paraformaldehyde (PFA; Merck) in PBS at 4°C for 30 minutes. Before labeling with the primary antibodies, cells were washed once with saponin buffer (0.01% saponin, Sigma-Aldrich,0.1% BSA in PBS), and blocked with 10% normal human serum in saponin buffer at RT for 30 minutes. Macrophages were then incubated with mouse anti-human CD40 (AbD Serotec, Oxford, UK) or CD206 (BD Biosciences, San Jose, CA, USA) at RT for one hour:. After washing with saponin buffer, cells were incubated with the secondary goat anti-mouse alexa fluor 488 antibody (Invitrogen) at 4°C for 30 minutes, and washed with saponin buffer before analysis by flow cytometry. As a negative control, only secondary antibody was used. Results are expressed as a fold change of mean fluorescence intensity (MFI) relative to untreated M0 macrophages.

### Actin staining

To analyze the subcellular localization of actin, different human macrophage subtypes were cultured in the presence of storage buffer (vehicle control) or 10 μg/ml recombinant human galectin-2 at day 7 on ibitreat chamber slides (Ibidi, Planegg/Martinsried, Germany) at a density of 1 x 10^6^ cells/ml in DMEM complete medium. Macrophages were fixed in 4% PFA in HBSS (Invitrogen) at RT for 10 minutes, and permeabilized with 0.1% Triton X-100 (Merck) in PBS at RT for 5 minutes. Finally, the cells were stained with 0.4 μg/ml phalloidin-tetramethyl rhodamine iso-thiocyanate (TRITC; Sigma-Aldrich) at RT for one hour. Imaging was performed by a confocal laser scanning microscope (Leica TCS SP2 AOBS, Leica Microsystems B.V., Rijswijk, The Netherlands). A total of five randomly selected fields at 63 times original magnification were acquired with Leica confocal software version 2.61 (Leica Microsystems, Wetzlar, Germany).

### Motility

Different macrophage subtypes at day 7 were cultured in the presence of storage buffer (vehicle control) or 10 μg/ml recombinant human galectin-2 on ibitreat chamber slides (Ibidi) at a density of 1 x 10^6^ cells/ml in DMEM complete medium.

The cells were recorded after one hour adhesion using an inverted time-lapse video microscope (Olympus IX81, Zoeterwoude, The Netherlands) housed in a 60% humidified, 5% CO_2_ gassed, temperature controlled (37°C) chamber. A total of four randomly selected fields were recorded for 24 hours every 12 minutes at a 20 times original magnification. The movies were converted to avi format with Cell^R software. WCIF Image J software (National Institutes of Health, Bethesda, Maryland, USA) was used to calculate the motility as measured by the travelled distance. Briefly, ten cells per microscopic field were tracked manually.

### Mouse models and tissue sampling

#### Whole blood cultures

All animal experiments were performed in compliance with Dutch government guidelines and principles of laboratory animal care (NIH Publication no. 85–23 revised 1985) and the Directive 2010/63/EU of the European Parliament. Animal experiments were approved by the animal welfare committee of the Leiden University Medical Center (LUMC). All animals were housed in the local animal facility of Leiden University Medical Center.

C57BL/6 mice (Charles River, Chatillon-sur-Chalaronne, France), CD14^-/-^ and TLR4^-/-^ mice (C57BL/6 background; The Jackson Laboratory, Bar Harbor, Maine, USA) were bred locally (LUMC). Blood samples were collected from the tail vein from six mice of each strain (male and female, 12–16 weeks old) and diluted 1:50 with RPMI 1640 (Invitrogen), supplemented with penicillin-streptomycin (PAA Laboratories, Cölbe, Germany) and glutamax (Invitrogen). Blood was incubated at 37°C in 5% CO_2_ overnight, in the presence of 100 ng/ml LPS (E. *coli* 0111:B4; Invivogen) or 10 μg/ml recombinant mouse galectin-2, in the absence or presence of 20 μg/ml PMB (Sigma-Aldrich). Cell-free supernatant was collected and TNFα level was measured by ELISA (BD Biosciences) according to the manufacturers protocol.

#### Immunofluorescence staining

For immunohistochemistry, we used C57BL/6 mice from a previous study.[11] Briefly, the left deep femoral artery was ligated just distal to the superficial and deep femoral artery bifurcation. The mice were sacrificed at day 7 after ligation and left adductor muscles were dissected, cryopreserved, sectioned (5 μm thick) with a HM-560 cryostat (Thermo Scientific, Runcorn, Cheshire, UK) and stored at -80°C until use.

Before staining, frozen sections were air dried for one hour. Available sections from 7 placebo-treated mice and 5 galectin-2-treated mice were fixed in acetone (10 min). After washing (PBS), sections were incubated with 10% NMS in 0.1% BSA/PBS for 30 minutes to block non-specific antibody binding, and stained with rat anti-murine F4/80 (1:100; AbD Serotec) for one hour. After washing, sections were incubated with goat anti-rat Alexa fluor 647 secondary antibody (Invitrogen) for one hour to identify macrophages. Next, sections were washed, blocked with 10% normal rat serum (NRS) in 1% BSA/PBS for 30 minutes, and incubated with both rabbit anti-murine alpha-smooth muscle actin (1:100; Abcam, Cambridge, UK) and rat anti-murine CD40 biotin (M1; 1:400; eBioscience, Vienna, Austria) or rat anti-murine CD206 biotin (M2; 1:50; Biolegend, London, UK) at 4°C overnight. After washing, sections were incubated for one hour with goat anti-rabbit Alexa fluor 488 secondary antibody (Invitrogen) to visualize smooth muscle cells or streptavidin Alexa fluor 555 (Invitrogen) to identify the two macrophage subsets. After washing, nuclei were counterstained with Hoechst (1:50.000; Invitrogen) for 3 minutes. Sections were photographed using Leica DM6000 microscope with LAS AF software, at 20 times original magnification. The ratio of CD40 to F4/80 or CD206 to F4/80-positive cells was quantified of defined collateral arteries in the adductor muscles in a blinded fashion.

### Statistical analysis

Intergroup comparisons were performed using Student's t-test (two-sided). Mann-Whitney U tests were used for group comparisons requiring nonparametric analytic approaches. P-values less than 0.05 were considered significant. All experiments were performed with at least three independent donors, and data are presented as mean ± standard error of the mean (SEM), unless otherwise indicated. All statistics were performed using GraphPad Prism version 6.0 (Graphpad Software, San Diego, CA, USA).

## Results

### Galectin-2 binds monocytes and macrophages through CD14 and activates TLR4 signaling

**Fig 1:**
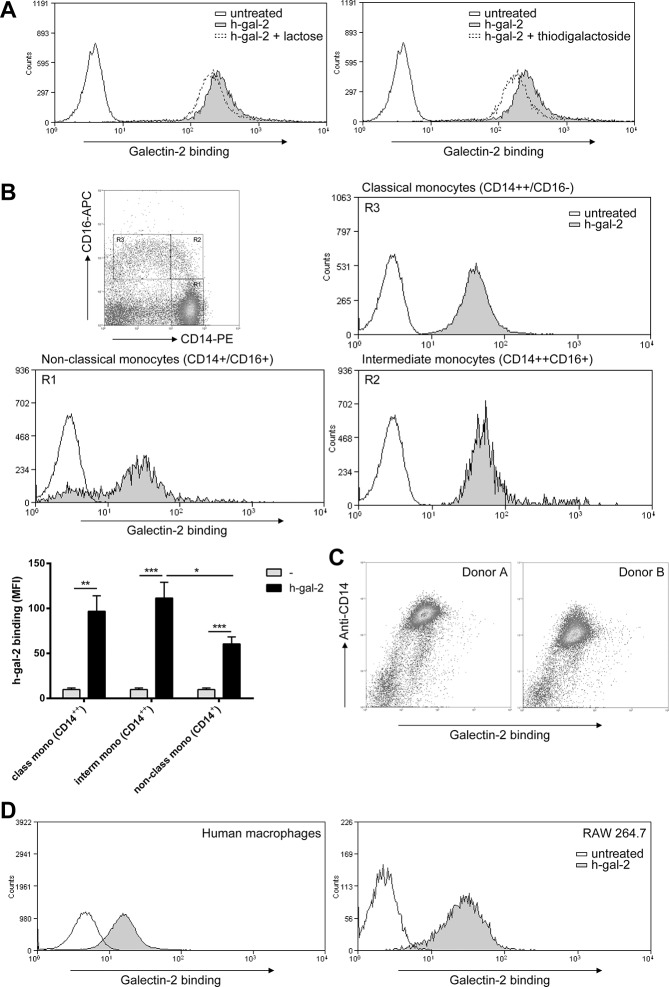
Binding analysis of recombinant human galectin-2 to monocytes and macrophages. Cells were incubated with 10 μg/ml biotinylated recombinant human galectin-2 (rh-gal-2). (A) The effect of lactose (20mM) and thiodigalactoside (20mM) on rh-gal-2 binding to monocytes. (B) Binding (expressed as MFI) of human galectin-2 to human monocyte subsets i.e. non-classical (gate R1; CD14+CD16+), intermediate (gate R2; CD14++/CD16+), and classical (gate R3; CD14++CD16-). Results are presented as mean ± SEM from at least 3 independent experiments. * *P* < 0.05, **P < 0.01, ***P < 0.001, untreated vs. h-gal-2. (C) Correlation of rh-gal-2 binding with CD14 expression. (D) Binding of rh-gal-2 to human monocyte-derived M0 macrophages and mouse macrophages (RAW264.7). Representative histograms from three independent experiments are depicted in all three panels.

We evaluated the binding of human and mouse biotinylatedgalectin-2 to the surface of human monocytes by flow cytometry. Human monocytes bound both human and mouse galectin-2 with a high affinity (apparent Kd of 1.1 μg/ml (77 nM) and 2.5 ug/ml, respectively, S2 Fig). Both galectins bound in a concentration dependent manner with near maximal binding at10 μg/ml and an apparent Bmax of 93.6 and80.7 for human and mouse galectin-2, respectively. By contrast, a much lower binding was observed for human galectin-1 at equal fluorescent labeling (apparent Kd 0.2 μg/ml, Bmax of 28.9). Next, the carbohydrate-binding specificity of human galectin-2 was determined by adding either lactose or thiodigalactoside together with human galectin-2 to human monocytes. These disaccharides did not inhibit binding of human galectin-2 (Fig 1A), suggesting that the binding is carbohydrate-independent. Interestingly, different human monocyte subsets (classical CD14^++^CD16^-^; intermediate CD14^++^CD16^+^; and non-classical CD14^+^CD16^+^monocytes) showed differential binding of human galectin-2 (Fig 1B). CD14^low^ monocytes (non-classical) showed a lower binding of human galectin-2 than CD14^high^ monocytes (classical and intermediate), reaching significance for the comparison with intermediate monocytes (Fig 1B). These results suggest that binding is associated with expression levels of CD14. Additionally, binding of human galectin-2 to different types of macrophages was investigated. As shown in Fig 1C, human monocyte-derived macrophages (M0) and mouse macrophages (RAW264.7 cells) bound human galectin-2.Human monocyte-derived dendritic cells (DCs), and human T-cells did not bind human galectin-2 (S3 Fig).

**Fig 2:**
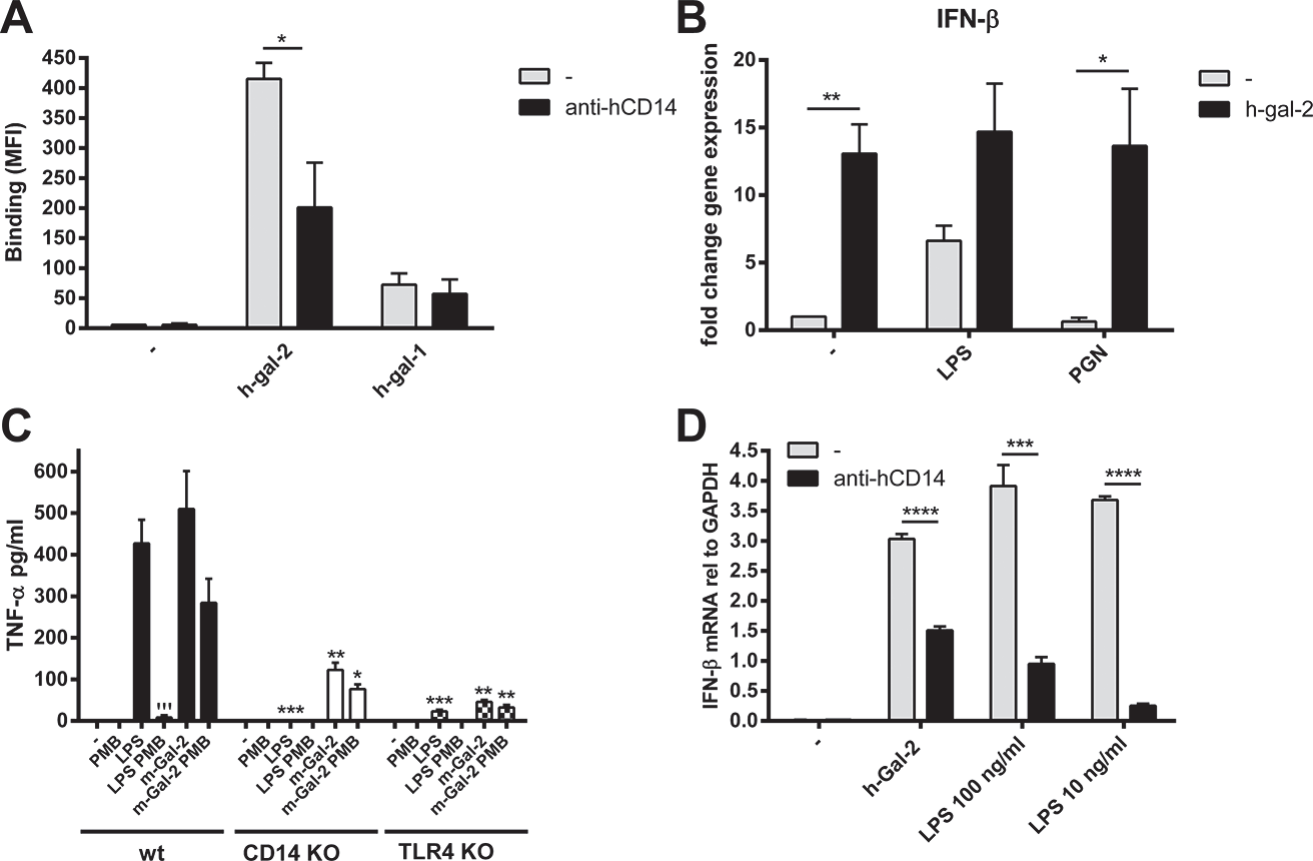
Galectin-2 binds CD14 and mimics LPS. (A) Human monocytes were incubated with biotinylated rh-gal-2 or—rh-gal-1. Binding of galectins (expressed as MFI) was analyzed by flow cytometry in the presence or absence of neutralizing anti-human CD14 antibody (100ug/ml). (B) IFN-β gene expression was measured in human monocytes by real-time PCR after treatment with TLR4 ligand LPS (10 ng/ml), TLR2 ligand PGN (1 μg/ml), rh-gal-2 (10 μg/ml), or combinations for three hours. Expression levels were compared of untreated vs. h-gal-2, LPS, and PGN. LPS vs. LPS + h-gal-2, and PGN vs. PGN + h-gal-2. Gene expression is expressed relative to untreated sample (set at 1). *P <0.05, **P < 0.01. Results in panel A and B are presented as mean ± SEM from at least 3 independent experiments. (C) Whole blood from WT, TLR4-/-, or CD14-/- mice was stimulated with rm-gal-2,or LPS in the absence or presence of polymyxin B (PMB). TNF-α protein levels were measured in supernatants and compared between wt and CD14- or TLR4-deficient cells stimulated as indicated. Within the mouse strains the effect of PMB was evaluated. *P < 0.05, **P < 0.01, ***P < 0.001, wt vs. knockout. '''*P* < 0.001, LPS vs. LPS + PMB. (D) Human monocytes were incubated with rh-gal-2 (10 μg/ml) or LPS (as indicated) for three hours in the presence or absence of neutralizing anti-human CD14 antibody (20 μg/ml).IFN-β gene expression was determined by real-time PCR. Results represent the mean expression ± SD of one experiment measured in triplicate. ***P < 0.001, ****P < 0.0001

Because of the association of galectin-2-monocyte binding with CD14 expression levels, we examined whether galectin-2 is a ligand for CD14.CD14 antibodies that neutralize activation of the CD14-associated TLR2 and TLR4 receptors [23,24] caused a 50% reduction in binding of human galectin-2 to human monocytes, whereas binding of human galectin-1 was not affected by these antibodies(Fig 2A).Next, we examined whether binding of galectin-2 to CD14 leads to TLR activation and the possible role of the two CD14-associated TLRs in activation. We analyzed whether galectin-2 is able to induce IFN-β gene expression. IFN-β activity is induced by the TLR4 ligand LPS, but not by the TLR2 ligand PGN (Fig 2B).[25]The observation that galectin-2 was able to induce IFN-β expression, comparable to LPS and irrespective of TLR2 stimulation, suggests that galectin-2 activates TLR4.To further explore this possibility, we stimulated mouse whole blood cells deficient for TLR4 or CD14 with m-galectin-2. Induction of TNF-α protein production was determined by ELISA, showing that both LPS and m-galectin-2 required CD14 and TLR4 for signaling (Fig 2C). The LPS response, but not the galectin-2-induced response was inhibited by polymyxin B. These results indicate that galectin-2 acts independent of LPS, and requires both CD14 and TLR4 to mediate its effects. Also in human monocytes, galectin-2 required CD14 for IFN-β induction. Neutralizing CD14 antibodies indeed inhibited the induction of IFN-β expression by approximately 50% (Fig 2D).

### Galectin-2 induces monocytes to express proinflammatory genes, inhibits the expression of pro-arteriogenic factors and inhibits monocyte migration

**Fig 3:**
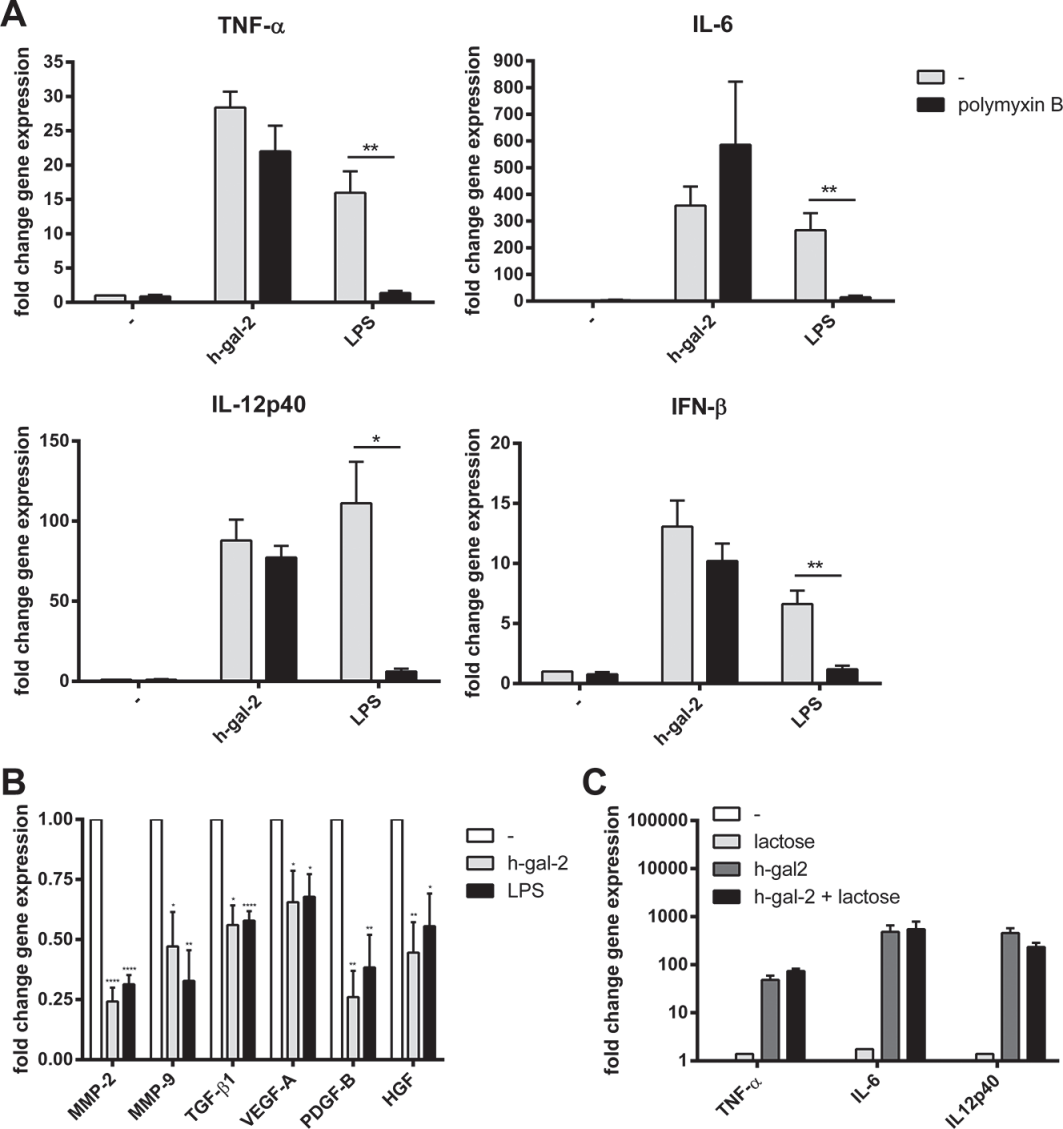
Human galectin-2 induces proinflammatory- and reduces proarteriogenic gene expression in monocytes. Human monocytes were incubated with rh-gal-2 or LPS for three hours in the presence or absence of PMB. Proinflammatory (A) and proarteriogenic (B) gene expression was determined by real-time PCR. (C) Lactose does not influence rh-gal-2-induced expression of proinflammatory genes, quantified relative to untreated sample (set at 1). Results represent mean ± SEM from at least 3 independent experiments. *P < 0.05, **P < 0.01, ****P< 0.0001.

To study the biological consequences of monocyte/galectin-2 interaction, we determined the effect of galectin-2 on pro- and anti-inflammatory gene expression. We observed a significant increase in the expression of proinflammatory genes such as TNF-α, IL-6, IL12-p40 and IFN-β upon incubation of monocytes with human galectin-2. In contrast to LPS-induced expression of these cytokines, their galectin-2 induced expression was not affected by polymyxin B (Fig 3A). Consistently, involvement of LPS in binding of galectin-2 to monocytes was excluded by FACS analysis, as binding was not affected by Polymyxin B (data not shown). These results show that the observed proinflammatory effects specifically result from galectin-2 and do not result from the presence of LPS contaminations in the galectin-2 preparations used.

Subsequently we studied the effect of galectin-2 on gene expression of known arteriogenic factors [26,26,27,27–38]. Monocytes stimulated with human galectin-2 showed a reduced gene expression of MMP-2 and -9, TGF-β1, VEGFA, PDGF-B, and HGF (Fig 3B). These effects were again comparable to the effects of LPS, indicating that galectin-2 indeed exert its effects via activation of the same CD14/TLR4 signaling pathway. Next, we evaluated whether the galectin-2-induced effects on gene expression were carbohydrate dependent. Effects of lactose on galectin-2 induced proinflammatory gene expression were not observed, which is in accordance with the lack of effect of lactose on galectin-2-monocyte binding and further confirms that the stimulatory effects of galectin-2 are not controlled by carbohydrate-dependent binding (Fig 3C).

**Fig 4:**
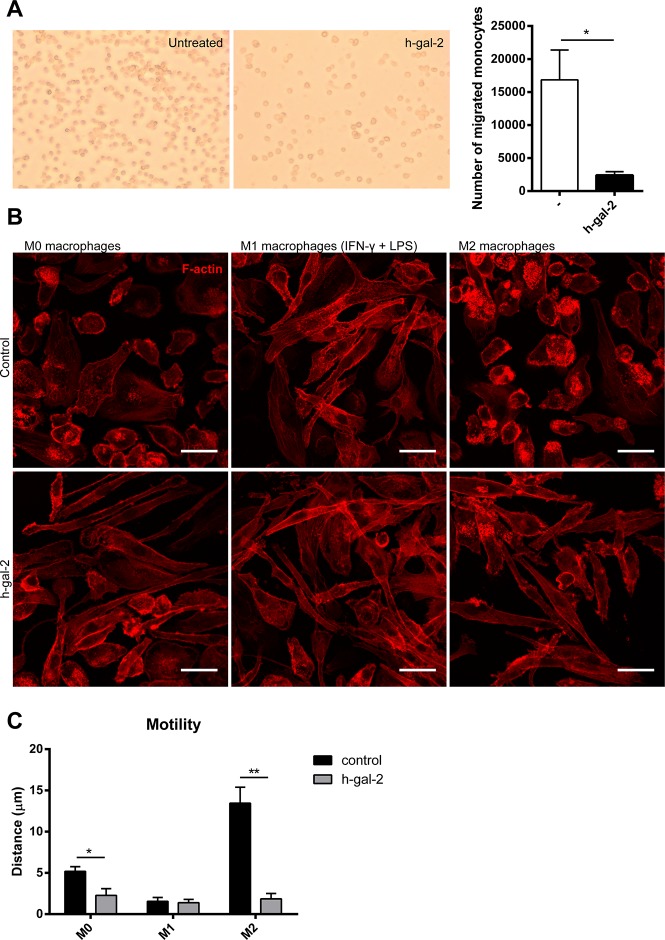
Human galectin-2 affects migration of monocytes and drives M1-type polarization of macrophages. (A) Spontaneous migration of human monocytes across fibronectin-coated Transwell inserts was assessed in the absence or presence of rh-gal-2. Representative pictures are shown of migrated cells after 24h. Quantified migrated cell counts represent the mean ± SEM from at least 3 independent experiments. *P < 0.05. (B) Human monocyte-derived differentiated macrophage subtypes (M0, M1 and M2) were stimulated at day 7 with either vehicle (control) or rh-gal-2 for 24 hours followed by actin staining (TRITC-labeled phalloidin). Representative images from three independent experiments are shown. A scale bar (25um) indicates the size of the cells, which become elongated (M1-like) in the presence of rh-gal-2. (C) Human monocyte-derived macrophage subtypes were stimulated at day 7 with either vehicle (control) or rh-gal-2. Motility is presented as mean traveled distance ± SEM, from at least 3 independent experiments. *P < 0.05, **P < 0.01.

Arteriogenesis depends on the migration of circulating monocytes into the vessel wall and local proliferation of tissue-resident macrophages [39,40]. Given the vital role of monocytes in arteriogenesis, we assessed the effect of human galectin-2 on human monocyte migration in a transwell system followed by flow cytometry. Galectin-2 treatment significantly inhibited monocyte migration (Fig 4A).

### Galectin-2 inhibits macrophage motility, induces macrophages to express M1 proinflammatory surface proteins and cytokines, and suppresses an M2 phenotype

Recent studies have shown a role for M2 macrophages in arteriogenesis.[14–16] Given the proinflammatory effect of galectin-2 on monocytes, we next hypothesized that galectin-2 might impair arteriogenesis by preventing pro-arteriogenic M2 macrophage differentiation Because M1 and M2 macrophages display distinct morphologies, associated with their differential migratory properties[41], we first studied the effect of human galectin-2 on the actin distribution of different human macrophage subtypes (M0, M1 [IFN-γ + LPS], and M2 [IL-4]). Galectin-2 treatment of M0 and M2 macrophages, which are typically round, caused an elongated cell shape, characteristic of M1 macrophages (Fig 4B). The LPS-induced elongated cell shape of M1 macrophages was not further affected by galectin-2. We have previously shown that M1 macrophages are less motile than M2 macrophages[41]. Therefore we assessed the effects of galectin-2 on macrophage motility by video time-lapse microscopy (S1 File, Videos 1–6). Galectin-2 treatment reduced the motility of M0 and M2 macrophages to a level comparable to M1 macrophages (Fig 4C).

**Fig 5:**
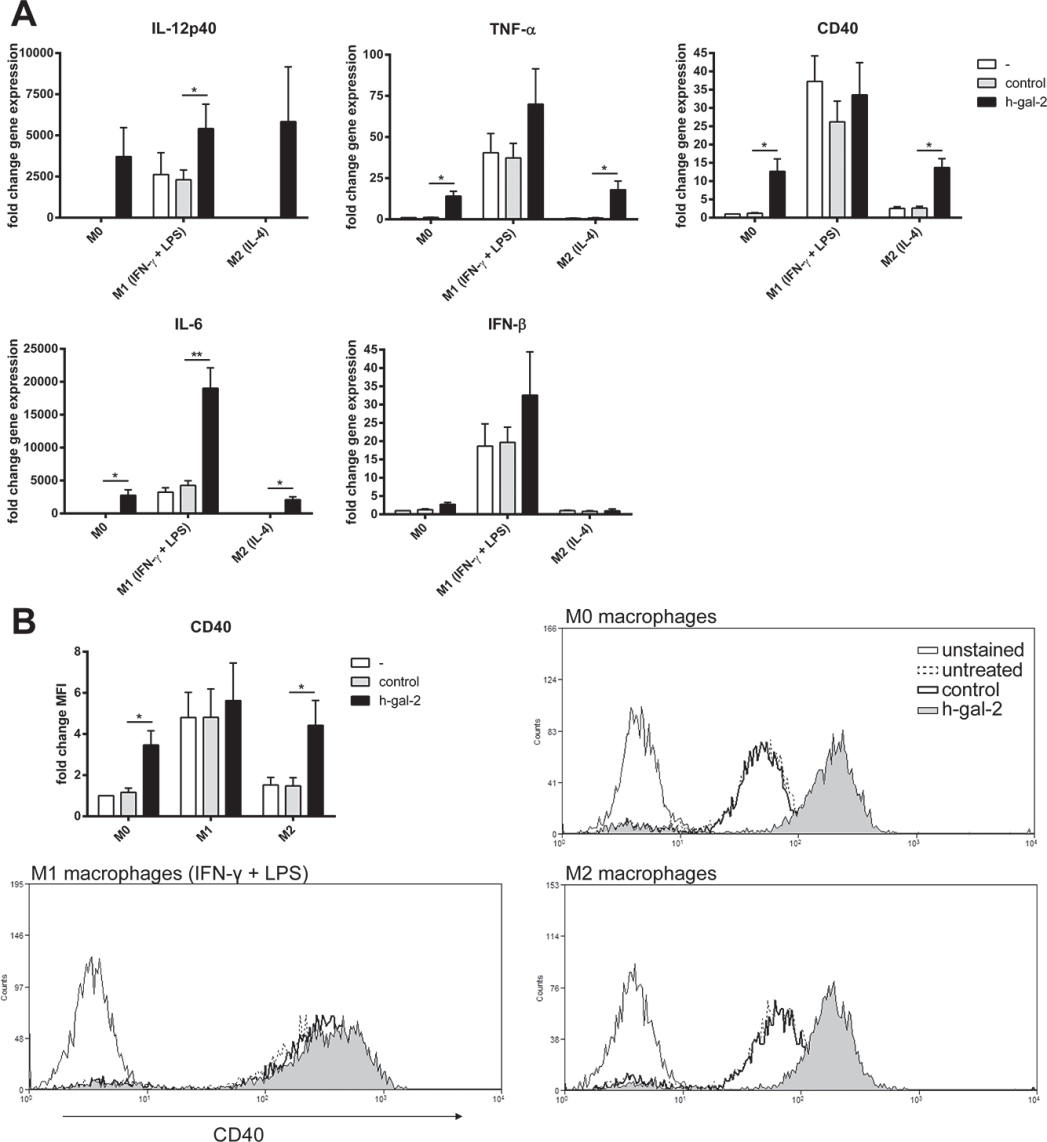
Human galectin-2 stimulation of macrophages results in gene transcription, and surface protein expression consistent with a polarized M1 phenotype. Differentiated human monocyte-derived macrophage subtypes were either left untreated (-), treated with vehicle (control) or rh-gal-2 for 24 hours. Macrophages were analyzed for the indicated M1 markers by real-time PCR (A) or flow cytometry (B). Representative histograms for CD40 expression are shown in panel B. Expression levels are expressed relative to M0 untreated samples (set at 1) as mean ± SEM from at least 3 independent experiments. *P < 0.05, **P < 0.01.

To assess the capacity of galectin-2 to induce a proinflammatory phenotype in the different macrophages subtypes, we studied gene expression of several M1 cytokines (IL12p40, TNF-α, IL-6, IFN-β)[17–21] and of the costimulatoryM1 moleculeCD40 by real-time PCR (RT-PCR)in M0, M1, and M2 macrophages. Human galectin-2 treatment induced gene expression of TNF-α, IL-6, CD40in M0 and M2 macrophages, and further increased the expression of IL-12p40 and IL-6 in M1 macrophages, while IFN-β expression was not affected (Fig 5A). Increased protein expression of CD40on M0 and M2 macrophages by galectin-2 was established using flow cytometry (Fig 5B). These results indicate that galectin-2 switches M0- and M2 macrophages towards a proinflammatoryM1 state, with surface protein expression of CD40.

**Fig 6:**
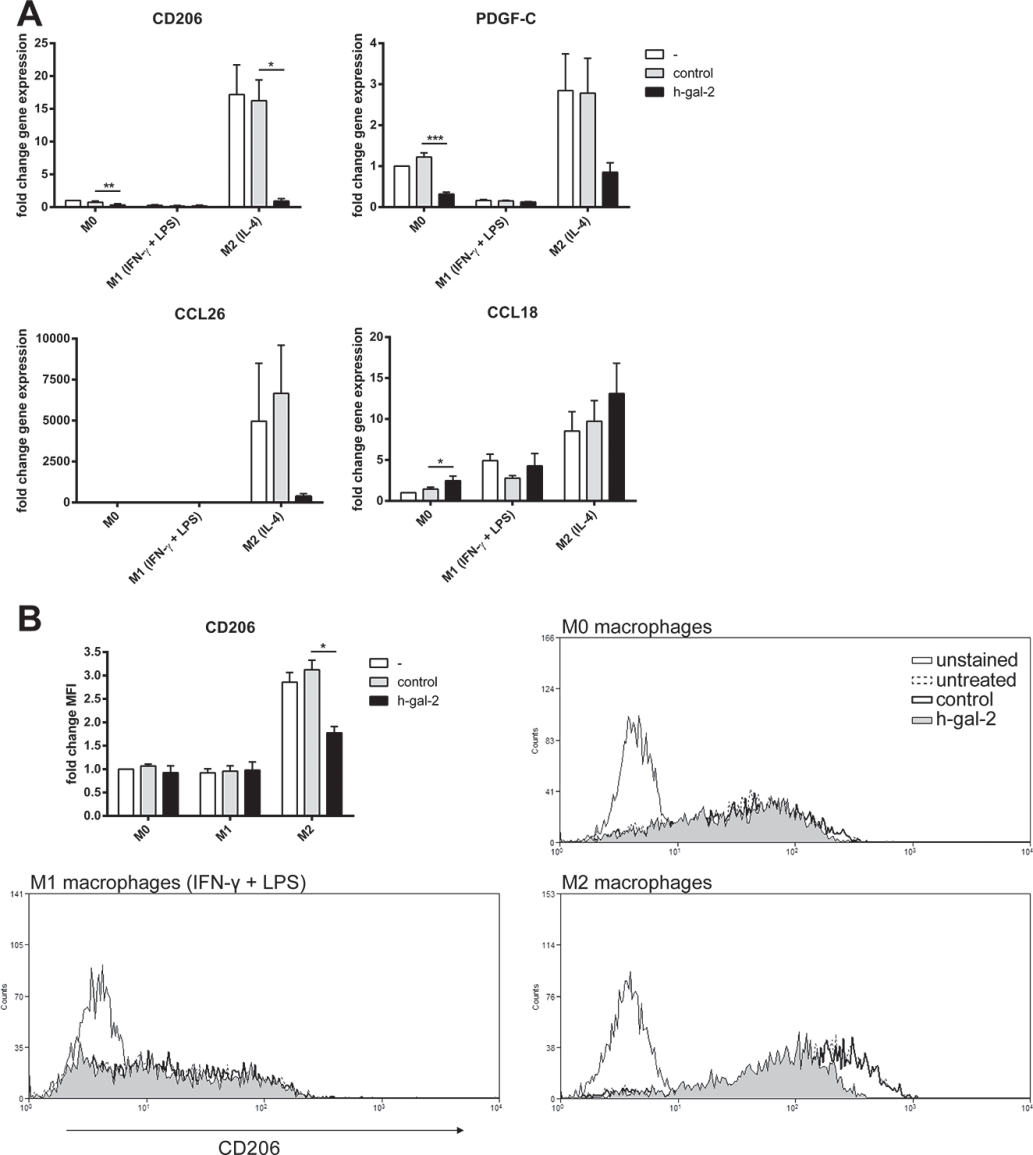
Human galectin-2 stimulation of macrophages does not affect gene transcription and surface protein expression of all M2 markers. Different human monocyte-derived macrophage subtypes at day 7 were stimulated as in Fig 5. Macrophages were analyzed for the indicated M2 markers by real-time PCR (A) or flow cytometry (B). Representative histograms for CD206 expression are shown in panel B. Expression levels are expressed relative to M0 untreated samples, as in Fig 5.

To test whether galectin-2 could reduce M2 markers, we measured the effects of galectin-2 on a set of distinctive M2-expressed marker genes[17–21]i.e. mannose receptor (CD206), PDGF-C, CCL26, and CCL18. RT-PCR data showed that galectin-2 significantly reduced mRNA expression of the mannose receptor by M0- and M2 macrophages, and reduced PDGF-C in M0macrophages (Fig 6A). Galectin-2 did not significantly inhibit the expression of the other M2 markers, CCL26 and CCL18. The reduced gene expression of the mannose receptor was confirmed at the protein level by flow cytometry (Fig 6B). Collectively, these data indicate that galectin-2 can partially modify differentiated M2 macrophages to M1 macrophages.

### Galectin-2 increases the number of M1 macrophages and reduces M2 macrophages around collateral arteries in vivo

**Fig 7:**
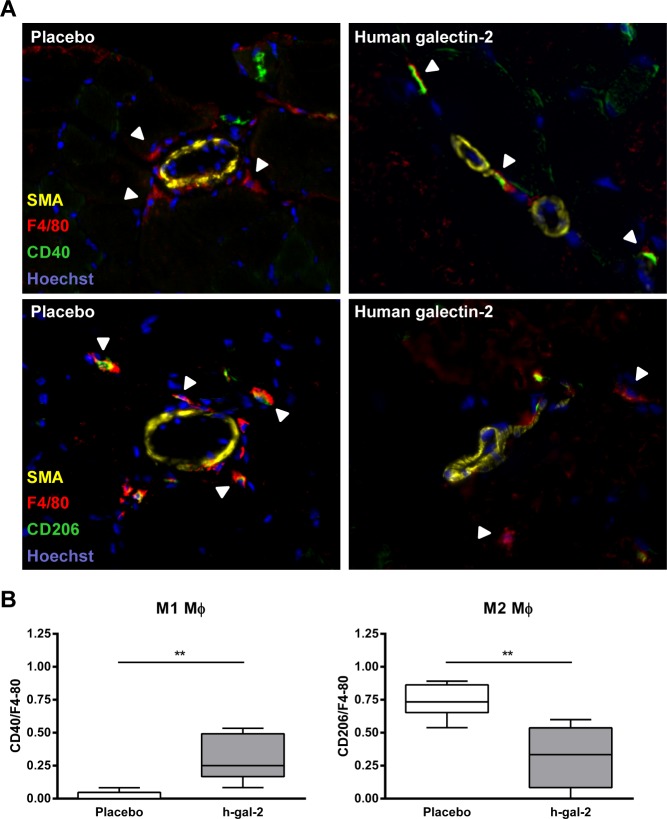
Human galectin-2 treatment induces M1 polarization of macrophages in vivo. (A) Representative immunofluorescence staining of CD40/F4-80 (M1) and CD206/F4-80 (M2) macrophages on muscle sections of the left adductor of the ligated hind limb obtained from placebo and human galectin-2 treated mice at 7 days after occlusion of the femoral artery is shown.Photomicrographs show arteries identified by SMA (yellow; Alexa fluor 488), cell nucleus by Hoechst (blue), macrophages by F4/80 (red; Alexa fluor 647) and macrophage subtypes by CD40 (green; streptavidin Alexa fluor 555) or CD206 (green; streptavidin Alexa fluor 555) staining as described in methods. Arrowheads indicate the macrophage subtypes. (B) Boxplot showing the counts of M1 and M2 macrophages in placebo vs. human galectin-2 treated mice. Data are presented as median and range. Boxes represent the first and third quartiles, the line within the box represents the median, and the lines outside the box represent the spread of the values. N = 7 for placebo, N = 5 for human galectin-2 treated group. (**P < 0.01, Mann-Whitney U test).

We previously showed that systemic administration of galectin-2 inhibits arteriogenesis after arterial ligation in the murine hind limb model [11]. Diminished arteriogenesis, as detected by Laser Doppler Perfusion Analysis and histology, was accompanied by a reduced number of macrophages surrounding the collateral vessels in the adductor muscle. We now used tissue sections from this study to assess the effect of human galectin-2 treatment on the presence of M1 and M2 macrophages around the collateral arteries *in vivo* by immunohistochemistry.M1-like macrophages were defined here as CD40^+^ F4/80^+^-positive cells while M2-like macrophages were detected as CD206^+^ F4/80^+^-cells, in close proximity to actively remodeling collateral arteries in the adductor muscle. This revealed that the number of M1 macrophages was significantly increased (24%) in mice treated with galectin-2 compared to placebo-treated mice. Furthermore, the number of M2 macrophages showed a significant decrease by 40% following galectin-2 treatment compared to placebo-treated mice (Fig 7). These results indicate that galectin-2 treatment in vivo promotes M1 and inhibits M2 macrophage accumulation during expansive arterial remodeling.

## Discussion

Galectin-2 expression in monocytes is associated with a low natural arteriogenic response in patients suffering from ischemic heart disease. *in vivo* data from a murine model of arteriogenesis showed that galectin-2 administration results in reduced arteriogenesis [11].

In the present study we describe that arteriogenesis can be affected by galectin-2 through modulation of monocyte/macrophage phenotype and physiology. Overall, we show that galectin-2 acts as a endogenous ligand that induces a proinflammatory status, in monocytes and macrophages, highly comparable to the effects of the well known exogenous modulator LPS.

We confirm and extend the previous finding that galectin-2 binds to monocytes[42], by showing its dose-dependency and, more importantly, that galectin-2 binding to human monocytes is carbohydrate-independent. Indeed, galectins may interact through protein-protein binding [1], as has been described earlier for the structurally related galectin-1 with H-ras [43]. The described affinity of galectin-2 and galectin-1 for oligosaccharides is in the micromolar range [44,45], whereas the affinity of galectin-2 for the surface of monocytes appeared to be in the nanomolar range. Together with the observed absence of an inhibiting effect of lactose on binding, this strongly indicates a highly specific protein-protein interaction. Consistent with this, we also did not observe any reduction in galectin-2-induced proinflammatory gene expression in human monocytes by lactose. This implies that also the effector functions of galectin-2 are independent of carbohydrate interaction.

Here, we provide several lines of evidence that galectin-2 requires CD14 for its binding to monocytes. CD14^high^ monocytes (i.e. classical and intermediate) show a higher binding to human galectin-2 than CD14^low^ monocytes (non-classical). Furthermore, immature DCs expressing low levels of CD14 [46] and T cells (CD14-negative) showed consistent lack of galectin-2 binding in our experiments. Finally, CD14 blocking antibodies reduced the binding of human galectin-2 to human monocytes.

CD14 is a known co-receptor for TLR4 and in rare instances also for TLR2 [23,24]. CD14 itself does not induce signaling, because it lacks a transmembrane part or cytoplasmic tail [47]. Signaling through CD14/TLR4 is MyD88-dependent, while the induction of IFN-β is MyD88-independent, and requires TRIF and IRF3 [23]. TLR2 signaling is also MyD88-dependent, but does not activate the TRIF-dependent pathway and therefore does not induce IFN-β expression. Galectin-2 strongly induced IFN-β gene expression in human monocytes to similar levels as LPS, indicating signaling through TLR4, although signaling through TLR2 is not entirely excluded. Our experiments with CD14- and TLR4-deficient murine cells confirmed that both CD14 and TLR4 are required for the NFκB-dependent induction of TNFα protein secretion by galectin-2.

Possible effects of LPS contamination were ruled out by including the LPS-neutralizing antibiotic polymyxin B as a control, at levels that completely blocked LPS responses. The possibility that galectin-2 would bind to TLR4 on monocytes via LPS was ruled out as well, by showing that polymyxin B, while preventing binding of LPS to macrophages [48], did not inhibit binding of galectin-2. Cytokine induction by galectin-2 was dependent on CD14, as established in two different models, being human cells in the presence of neutralizing CD14 antibodies and murine CD14-or TLR4-deficient cells.

Incubation of human monocytes with human galectin-2 led to increased expression of proinflammatory genes (IL-6, TNF-α, IL-12p40 and IFN-β), decreased expression of the anti-inflammatory and proarteriogenic cytokine TGFβ1 [49] and decreased expression of proarteriogenic factors MMP2, MMP9, VEGF-A, PDGF-B and HGF. These effects establish a key role for galectin-2 in monocyte proinflammatory activation status. Galectin-2 also bound human M0 macrophages and stimulated their polarization to an M1-like phenotype, as evidenced by a characteristic elongated cell shape, reduced motility and induction of proinflammatory expression of surface proteins and cytokines. These findings were corroborated by a highly increased surface protein expression of the proinflammatory M1 marker CD40. In contrast to M0 and M2 macrophages, M1 macrophages do not form filopodia upon stimulation with a chemoattractant, which may explain their reduced motility and migration relative to M0 and M2 macrophages [41]. An inhibiting effect of galectin-2 on monocyte migration has been described before [42]. Here, we confirm and extend these observations, showing that migration and motility of monocytes as well as M0 -, and M2 macrophages were almost completely blocked by galectin-2. Galectin-2 stimulation of M2 macrophages also resulted in a change from a round- to an elongated cell shape accompanied by a markedly reduced motility and a reduced expression of the M2-specific marker CD206, at both mRNA and protein level.

Together, these data show that galectin-2 polarizes monocytes and macrophages to a proinflammatory M1 state, while preventing pro-arteriogenic M2 differentiation.

These *in vitr*o results were further substantiated by our observation that galectin-2 treatment increased the number of M1-like, CD40-positive macrophages and reduced the number of M2-like CD206-positive macrophages in close proximity of actively expanding collateral arteries in a murine model for arteriogenesis.

In summary, galectin-2 is shown to be an endogenous ligand for CD14 on human monocytes, responsible for inducing a proinflammatory phenotype in monocytes and macrophages by provoking TLR4-dependent signaling. Targeting of galectin-2-mediated responses in monocytes and macrophages may provide new therapeutic strategies in coronary artery disease patients with a low arteriogenic response and a high expression of galectin-2.

## Supporting Information

S1 Fig SDS-PAGE analysis of the purified recombinant galectin proteins. Electrophoresis wasconducted on a 15% polyacrylamide gel under reducing conditions, and protein bands werevisualized by Coomassie blue staining. *Lanes 1*, *2 and 3* depict affinity-purified recombinanth-gal-2, m-gal-2, and h-gal-1, respectively. Molecular mass standards are indicated on the *left*. The h-gal-2 and m-gal-2 correspond to a protein band at about 15 kDa, and h-gal-1exists as a dimer at about 28 kDa.
(TIF)

S2 Fig Binding analysis of recombinant galectin proteins to human monocytes. Monocytes were stimulated with 0, 0.1, 0.5, 1, 2.5, 5, 10, and 20 μg/ml biotinylated recombinant galectin proteins at 4°C for 30 minutes, followed by strept avidin-alexa fluor 488 incubation at 4°C for 30 minutes, and binding (expressed as MFI) was analyzed by flow cytometry. Values for the *Kd* and *Bmax* were calculated from the untransformed data using the following equation, Binding = (Bmax x [galectin])/(Kd + [galectin]) using Graphpad Prism version 6.0. Dotted line indicates binding of h-gal-2 (black), h-gal-1 (dark grey), and m-gal-2 (light grey).
(TIF)

S3 Fig Binding of human galectin-2 to human immune cells. Immature human monocyte-derived dendritic cells, and human T-cells were incubated with 10 μg/ml biotinylated recombinant human galectin-2 at 4°C for 30 minutes, followed by streptavidin-alexa fluor 488 incubation at 4°C for 30 minutes, and binding was assessed by flow cytometry. Open histograms indicate background staining in the absence of galectin-2, grey histograms indicate galectin-2 binding.
(TIF)

S1 File Video files: recombinant human galectin-2 induces the M1 phenotype in macrophages. Human monocyte-derived macrophage subtypes were stimulated at day 7 with vehicle (control) or 10 μg/ml recombinant human galectin-2, and morphological changes and movement were recorded by an inverted time-lapse video microscope (Olympus IX81) for 24 hours. Experiment is performed at 60% humidity, 37°C in 5% CO_2_ and recorded with a 20x objective lens. The movies were converted to avi format with Cell^R software. Bar represents 200 μm. **Video 1:** M0 macrophages stimulated with vehicle control. **Video 2:** M0 macrophages stimulated with rh-gal-2. **Video 3:** M1 macrophages stimulated with vehicle control. **Video 4:** M1 macrophages stimulated with rh-gal-2. **Video 5:** M2 macrophages stimulated with vehicle control. **Video 6:** M2 macrophages stimulated with rh-gal-2.
(ZIP)
