# Supplementary figures and images for: Galectin-2 Induces a Proinflammatory, Anti-Arteriogenic Phenotype in Monocytes and Macrophages
Source: PLoS One. 2015 Apr 17;10(4):e0124347. doi: 10.1371/journal.pone.0124347 (PMC4401781; doi:10.1371/journal.pone.0124347)

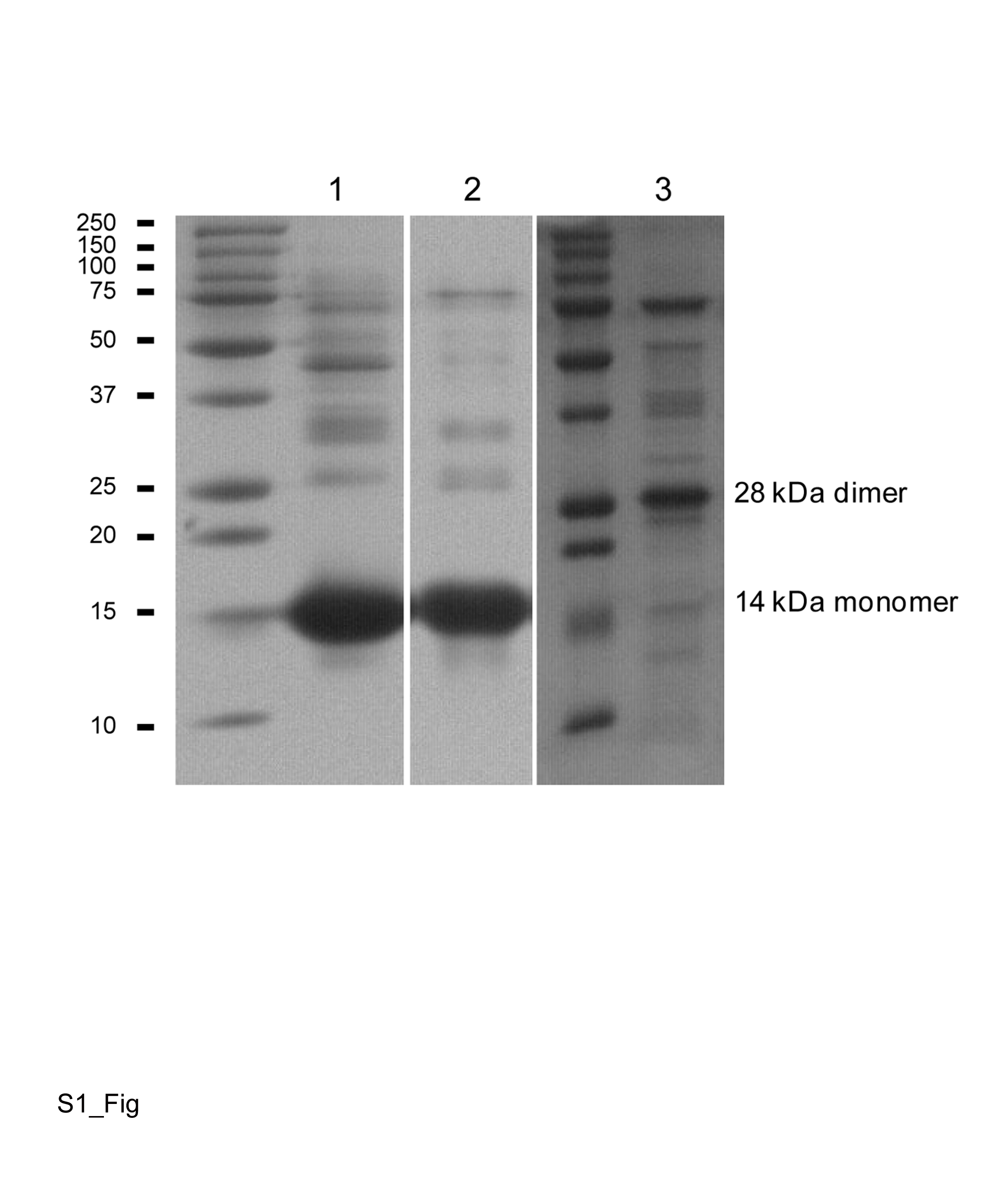

Supplement: S1 Fig — Electrophoresis wasconducted on a 15% polyacrylamide gel under reducing conditions, and protein bands werevisualized by Coomassie blue staining. Lanes 1, 2 and 3 depict affinity-purified recombinanth-gal-2, m-gal-2, and h-gal-1, respectively. Molecular mass standards are indicated on the left. The h-gal-2 and m-gal-2 correspond to a protein band at about 15 kDa, and h-gal-1exists as a dimer at about 28 kDa. (TIF) [file pone.0124347.s001.tif]

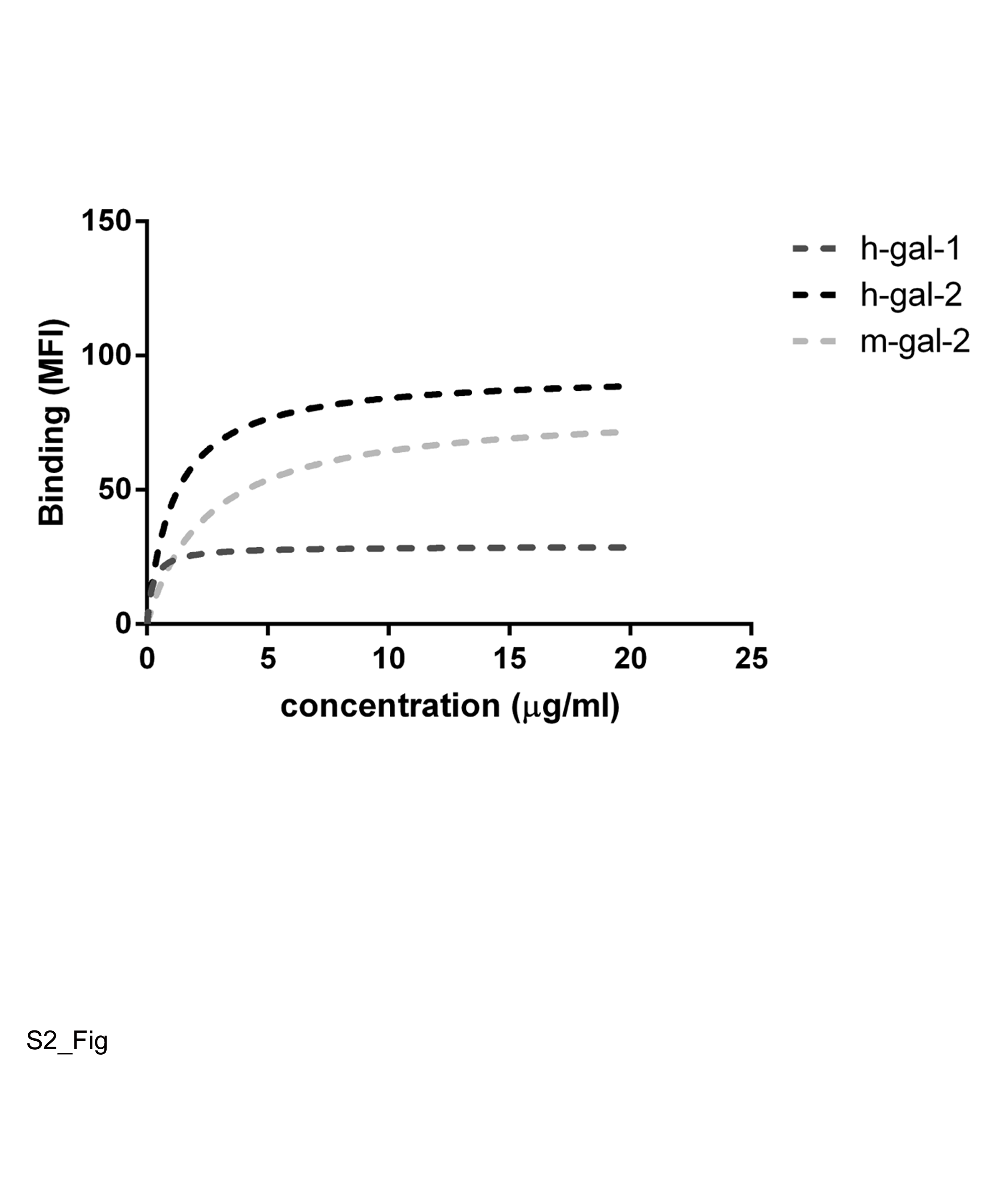

Supplement: S2 Fig — Monocytes were stimulated with 0, 0.1, 0.5, 1, 2.5, 5, 10, and 20 μg/ml biotinylated recombinant galectin proteins at 4°C for 30 minutes, followed by strept avidin-alexa fluor 488 incubation at 4°C for 30 minutes, and binding (expressed as MFI) was analyzed by flow cytometry. Values for the Kd and Bmax were calculated from the untransformed data using the following equation, Binding = (Bmax x [galectin])/(Kd + [galectin]) using Graphpad Prism version 6.0. Dotted line indicates binding of h-gal-2 (black), h-gal-1 (dark grey), and m-gal-2 (light grey). (TIF) [file pone.0124347.s002.tif]

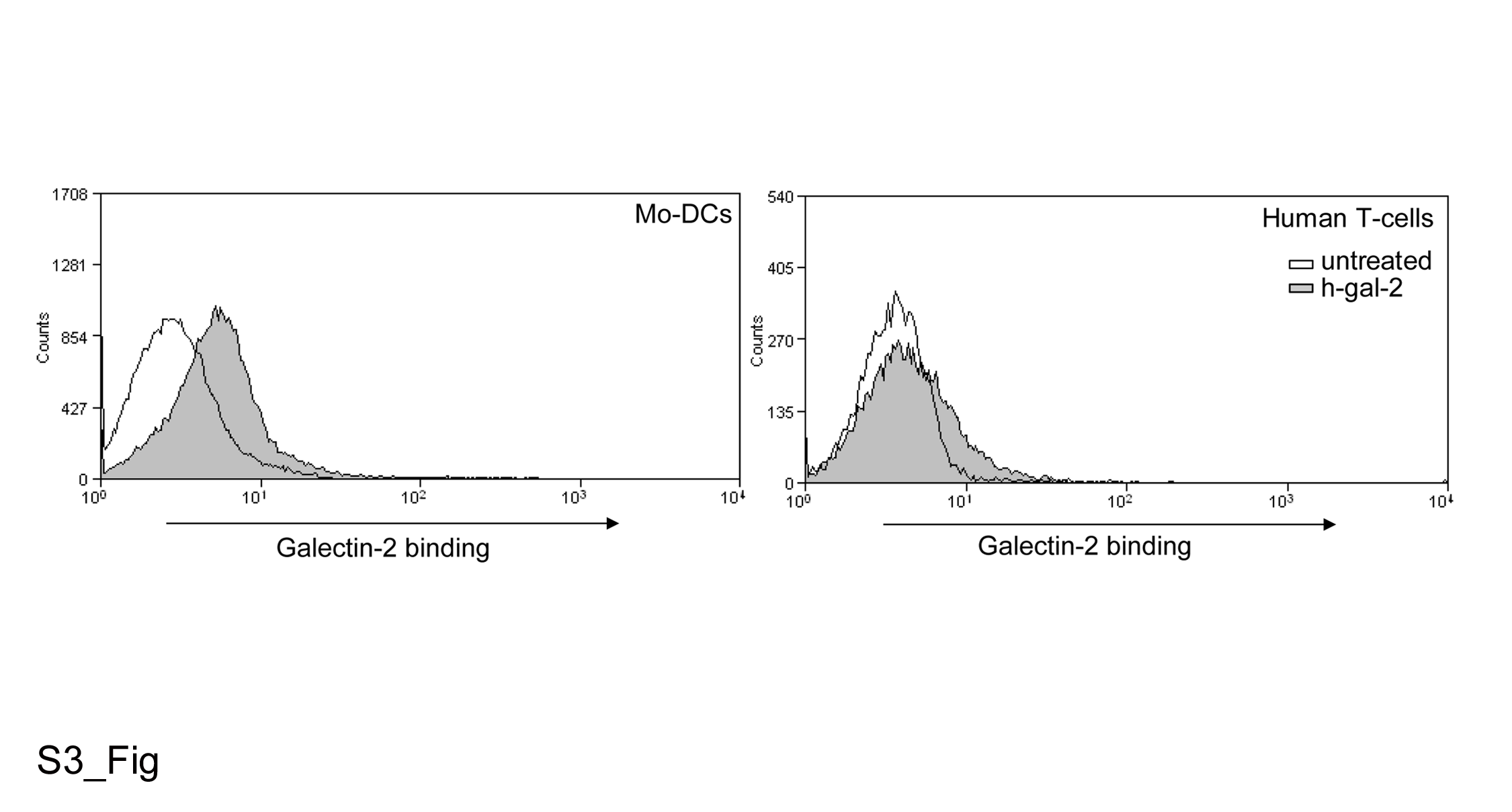

Supplement: S3 Fig — Immature human monocyte-derived dendritic cells, and human T-cells were incubated with 10 μg/ml biotinylated recombinant human galectin-2 at 4°C for 30 minutes, followed by streptavidin-alexa fluor 488 incubation at 4°C for 30 minutes, and binding was assessed by flow cytometry. Open histograms indicate background staining in the absence of galectin-2, grey histograms indicate galectin-2 binding. (TIF) [file pone.0124347.s003.tif]
